# Prevalence and Genetic Characterization of Two Mitochondrial Gene Sequences of Strobilocercus Fasciolaris in the Livers of Brown Rats (*Rattus norvegicus*) in Heilongjiang Province in Northeastern China

**DOI:** 10.3389/fcimb.2020.588107

**Published:** 2020-11-25

**Authors:** Fengnian Zhao, Yun Zhou, Yanchen Wu, Kexin Zhou, Aiqin Liu, Fengkun Yang, Weizhe Zhang

**Affiliations:** Department of Parasitology, Harbin Medical University, Harbin, China

**Keywords:** strobilocercus fasciolaris, *Hydatigera taeniaeformis*, brown rats, prevalence, genetic characterization

## Abstract

Rodents constitute the largest and most successful group of mammals worldwide. Brown rats (*Rattus norvegicus*) are one of the most common rodent species, and they serve as intermediate hosts of *Hydatigera taeniaeformis.* Although there have been a few studies reporting on the presence of the larval form of *H. taeniaeformis* (strobilocercus fasciolaris) in brown rats worldwide, little information is available on the genetic characterization of this parasite, with no molecular data from China. Therefore, from April 2014 to March 2016, this study was carried out to understand the prevalence and genetic characters of strobilocercus fasciolaris in brown rats captured in Heilongjiang Province in northeastern China. The livers of brown rats were collected and examined for the presence of cysts. Each cyst was identified based on morphological observation: the larvae with the naked eye and the scolexes under a microscope. The results were confirmed by polymerase chain reaction (PCR) and sequencing of the cytochrome *c* oxidase subunit 1 (*cox1*) and NADH dehydrogenase subunit 4 (*nad4*) genes. At the investigated sites, 11.8% (13/110) of the brown rats were infected with strobilocercus fasciolaris. Based on sequence analysis, there were 10 and six haplotypes regarding the *cox1* and the *nad4* loci, with 24 and 42 polymorphic sites, respectively (degree of intraspecific variation: 0.3%–4.4% and 0.6%–4.7%, respectively). Twelve nucleotide sequences (six of the 10 at the *cox1* locus and all six at the *nad4* locus) have not previously been described. Base differences in three of the six novel *cox1* gene sequences and five of the six novel *nad4* gene sequences caused amino acid changes. Phylogenetic analyses of the *cox1* and *nad4* gene sequences based on neighbor-joining and Bayesian inference trees indicated that all the strobilocercus fasciolaris isolates belonged to *Hydatigera taeniaeformis* sensu stricto (s.s.). This is the first report on the genetic characterization of strobilocercus fasciolaris in brown rats in China. The findings of novel *cox1* and *nad4* nucleotide and amino acid sequences may reflect the region-specific genetic characterization of the parasite. The data will be useful to explore the biological and epidemiological significance of the intraspecific variation within *H. taeniaeformis* s.s.

## Introduction

Taeniidae is one of the most important families of the order Cyclophyllidea, which contains most of the zoonotic parasites of medical significance. Some members of the genus *Taenia* are responsible for taeniasis and/or cysticercosis in humans (Sharma et al., 2016). The resurrection of the genus *Hydatigera* was proposed in a recent revision of the family Taeniidae; *Hydatigera* consists of four valid species, *Hydatigera taeniaeformis* sensu stricto (s.s.), *Hydatigera krepkogorski*, *Hydatigera parva*, and *Hydatigera kamiyai* (Nakao et al., 2013a; Nakao et al., 2013b; Catalano et al., 2019). *H. taeniaeformis* is found in the small intestine of cats and other felids, which are the definitive hosts. They acquire the parasitic infection by consuming the livers of rats and mice (the intermediate hosts) infected with the larval form of *H. taeniaeformis* (strobilocercus fasciolaris) (Singla et al., 2008; Moudgil et al., 2016). Brown rats (*Rattus norvegicus*) are one of the most widely known and most common rat species. Natural infections involving strobilocercus fasciolaris have been reported in brown rats in many countries/regions (Sharma et al., 2017). To date, several human cases have been documented: strobilocercus fasciolaris infection in the liver of a 77-year-old man from Czechoslovakia (Sterba and Barus, 1976) and *H. taeniaeformis* infection in the small intestine of individuals from Argentina, Japan and Sri Lanka (Sterba and Barus, 1976; Ekanayake et al., 1999; Hoberg, 2002).

The use of polymerase chain reaction (PCR)-based molecular tools has contributed to accurate species differentiation and a better understanding of the genetic characterization of *H. taeniaeformis*. As mitochondrial (mt) DNA is known to have a faster evolutionary rate than nuclear DNA, mt genes have been widely used to identify taeniid species and strains and to assess the genetic relationships among them (Okamoto et al., 1995; Dai et al., 2012). However, there is limited information on the genetic variation and phylogenetic relationships regarding *H. taeniaeformis* population worldwide (Lavikainen et al., 2016). In China, *H. taeniaeformis* adults have been found in cats (Xu et al., 1994; Lin et al., 1995; Wang et al., 1995; Wang et al., 1997), while the larvae have been found in rats and mice (based on morphological analysis), including brown rats (11.6%–53.6%) (Wu and Yin, 2005; Tan et al., 2008), buff-breasted rats (*Rattus flavipectus*) (3.3%–38.0%) (Huang, 1991; Yuan et al., 2000), lesser rice-field rats (*Rattus losea*) (16.3%) (Yuan et al., 2000), black rats (*Rattus rattus*) (15.0%) (Huang, 1991), and house mice (*Mus musculus*) (16.2%) (La and Zhao, 1989). However, there are no available reports on the genetic characterization of strobilocercus fasciolaris in brown rats in China. Therefore, we carried out this epidemiological study of the parasite in brown rats in Heilongjiang Province, northeastern China, and conducted a genetic characterization of the isolates.

## Materials and Methods

### Study Sites and Collection of Rats

From April 2014 to March 2016, 110 brown rats were captured in cage traps baited with sunflower seeds and peanut/sesame butter in four areas in Heilongjiang Province, northeastern China: 23 in a granary in Xingren Town, 27 in a pig farm in Mingshui County, 27 in a pig farm in Qinggang County, and 33 in a sheep farm in Baoqing County. All the captured rats were euthanized by CO_2_ inhalation and transported to our laboratory in coolers with ice packs. Procedures involving the rats were strictly conducted according to the Chinese Laboratory Animal Administration Act of 1998.

### Collection of Liver Samples and Examination of Cysts

After euthanasia, the liver was collected from each rat and examined for the presence of cysts. Each cyst was opened by making a small slit. If a single larva was released with liquid, and a large scolex could be observed with four lateral suckers and double rows of hooks using a light microscope at 400× magnification, the larva was suspected to be strobilocercus fasciolaris. They were preserved in 70% ethanol at 4°C for further molecular analysis.

### Extraction of Genomic DNA

Prior to DNA extraction, each parasite specimen was washed three times with phosphate-buffered saline to remove the ethanol. Parasitic genomic DNA was extracted from approximately 25 mg of each larva using a DNeasy Blood & Tissue Kit (Qiagen, Hilden, Germany) according to the manufacturer’s instructions. DNA was eluted in 200 µl of AE elution buffer (provided with the kit) and stored at −20°C until further PCR analysis.

### PCR Amplification

The partial cytochrome *c* oxidase subunit 1 (*cox1*) and NADH dehydrogenase subunit 4 (*nad4*) genes (approximately 450 and 660 bp, respectively) of *H. taeniaeformis* were amplified using the primers and protocols described previously by Bowles and McManus (1994) and Dai et al. (2012), respectively. TaKaRa Taq DNA polymerase (TaKaRa Bio Inc., Tokyo, Japan) was used for all PCR amplifications. Sterile deionized water served as the negative control. The PCR products were subjected to electrophoresis in 1.5% agarose gel and visualized by staining the gel with GelStain (TransGen Biotech, Beijing, China).

### DNA Sequencing and Molecular Analysis

The PCR products were then sequenced with PCR primers for each gene on an ABI PRISM 3730 XL DNA Analyzer (Applied Biosystems, Carlsbad, CA, USA) using a Big Dye Terminator v3.1 Cycle Sequencing kit (Applied Biosystems). The accuracy of the sequencing data was confirmed by sequencing the PCR products from forward and reverse directions. The nucleotide sequences were then used in Basic Local Alignment Search Tool (BLAST) searches (http://www.ncbi.nlm.nih.gov/blast.cgi). They were aligned with each other and with reference sequences that were downloaded from GenBank using ClustalX v1.83 (http://www.clustal.org/).

### Phylogenetic Analysis

To explore the genetic and geographical relationships of *H. taeniaeformis* isolates, phylogenetic analyses of the *cox1* and *nad4* gene sequences were performed using two common phylogenetic methods: the neighbor-joining method with MEGA v6.0 (http://www.megasoftware.net) and Bayesian inference with MrBayes v3.2.6 (http://phylosuite.jushengwu.com/). For the countries for which there were more than two *H. taeniaeformis* sequences in GenBank, the two sequences with the largest base difference were selected for each locus being analyzed. However, for some countries, we only used one sequence, as there was only one sequence deposited in GenBank. The reliability of the neighbor-joining trees was assessed using bootstrap analysis with 1000 replicates, and the evolutionary distances were calculated using the Kimura-2-parameter model. The Jukes–Cantor model was used for Bayesian inference. Bayesian posterior probability values were determined after running the Markov chains (two runs, four chains) for 2 million generations and discarding the first 25% of samples as burn-in. The consensus tree was rooted at its midpoint and visualized using FigTree v1.4.2 (http://tree.bio.ed.ac.uk/software/figtree/).

## Results and Discussion

Thirteen of the 110 brown rats (11.8%) were confirmed to be infected with strobilocercus fasciolaris in the livers based on morphological observation (the larvae in the cysts with the naked eye and the scolexes under a microscope) and by sequence analysis of the partial *cox1* and *nad4* genes. The prevalence of 11.8% was lower than that reported in brown rats in the Philippines (100%) (Claveria et al., 2005), India (36.0%) (Singla et al., 2008), Korea (33.8%) (Lee et al., 2016), Serbia (29.9%) (Kataranovski et al., 2010), and Grenada, West Indies (29.6%–67.6%) (Chikweto et al., 2009; Sharma et al., 2017). The prevalence has been reported to be in the range of 11.6%–53.6% in another six Chinese provinces (Wu and Yin, 2005; Tan et al., 2008). The prevalence is complex and related to many factors. For example, regarding the rat age, 25–60-day-old albino rats were observed to be more susceptible to the parasite than younger or older albino rats in a study of age-related resistance to strobilocercus fasciolaris (Greenfield, 1942). However, Lee et al. (2016) pointed out that the prevalence was more closely related to infection accumulation with age, rather than host age-dependent differences in susceptibility to the parasite, and host body weight was positively associated with the prevalence. Additionally, Sharama et al. (2017) believed that human population density influenced the prevalence of strobilocercus fasciolaris in brown rats in the two study sites in Grenada, West Indies.

By comparing the *cox1* and *nad4* gene sequences of the 13 isolates, 10 and six haplotypes were found, with 24 and 42 polymorphic sites being observed, respectively; the degree of intraspecific variation was 0.3%–4.4% (1–18 base differences) at the *cox1* locus and 0.6%–4.7% (4–31 base differences) at the *nad4* locus (**Tables 1**, **2**). Intraspecific variation within *H. taeniaeformis* has been described previously. In 2016, 150 specimens of *Hydatigera taeniaeformis* sensu lato (s.l.) from various definitive and intermediate hosts in Eurasia, Africa and Australia were analyzed and a new species, *H. kamiyai*, was separated from *H. taeniaeformis* s.l. (Lavikainen et al., 2016). This finding supported the earlier discovery in 1995 of a presumed novel species, the TtACR isolate, from the grey red-backed vole (*Myodes rufocanus bedfordiae*) in Japan (Okamoto et al., 1995). The TtACR isolate was observed to have a degree of variation of 9.0%–9.5% at the *cox1* locus compared to isolates from other murine species including brown rats in Japan (n = 1) and Malaysia (n = 1), house mice in Belgium (n = 1) and China (n = 1), and small Japanese field mice (*Apodemus argenteus*) in Japan (n = 2) (Okamoto et al., 1995). In 2008, an analysis of the *cox1* locus showed that a Turkish isolate (TtaTu) from a wood mouse (*Apodemus sylvaticus*) and a Finnish isolate (TtaFi) from a cat (*Felis catus*) were genetically close to the divergent isolate from Japan (TtACR), and the three isolates were genetically distant from a Kazakhstan isolate (TtaKa) from a wood mouse (which had a *cox1* gene sequence that resembled the majority of the previously published *cox1* gene sequences of *H. taeniaeformis*) (Lavikainen et al., 2008). Variation has been clearly observed at the *cox1* locus: 0.3%–5.1% within *H. taeniaeformis* s.s., and 9.1%–13.3% between *H. taeniaeformis* s.s. and *H. kamiyai* (including the TtACR, TtaFi and TtaTu isolates) (Lavikainen et al., 2016). Due to the rarity of data on the *nad4* gene sequences of *H. taeniaeformis* from other countries/regions, we did not conduct a corresponding comparative analysis in this study.

**Table 1:** Values of nucleotide variation in the *cox1* gene detected between pairs of strobilocercus fasciolaris sequences, expressed as percentages.

Accession no.	MF380373	MF380374	MF380375	MF380376	MF380377	MF380378	MF380379	MF380380	MF380381	MF380382
MF380373	―									
MF380374	0.5	―								
MF380375	1.0	1.5	―							
MF380376	1.2	1.7	0.3	―						
MF380377	1.8	1.9	0.5	0.7	―					
MF380378	2.5	2.9	3.2	3.4	3.7	―				
MF380379	1.5	1.9	1.2	1.5	1.7	3.7	―			
MF380380	0.3	0.7	1.2	1.5	1.7	2.7	1.7	―		
MF380381	1.2	1.7	0.3	0.5	0.3	3.4	1.5	1.5	―	
MF380382	3.2	3.7	1.2	4.2	4.4	0.7	3.9	3.4	4.2	―

**Table 2:** Values of nucleotide variation in the *nad4* gene detected between pairs of strobilocercus fasciolaris sequences, expressed as percentages.

Accession no.	MF380383	MF380384	MF380385	MF380386	MF380387	MF380388
MF380383	―					
MF380384	2.3	―				
MF380385	4.7	4.0	―			
MF380386	2.1	1.4	4.1	―		
MF380387	0.6	2.0	4.7	1.8	―	
MF380388	4.7	4.0	0.6	4.1	4.7	―

**Figure 1:**
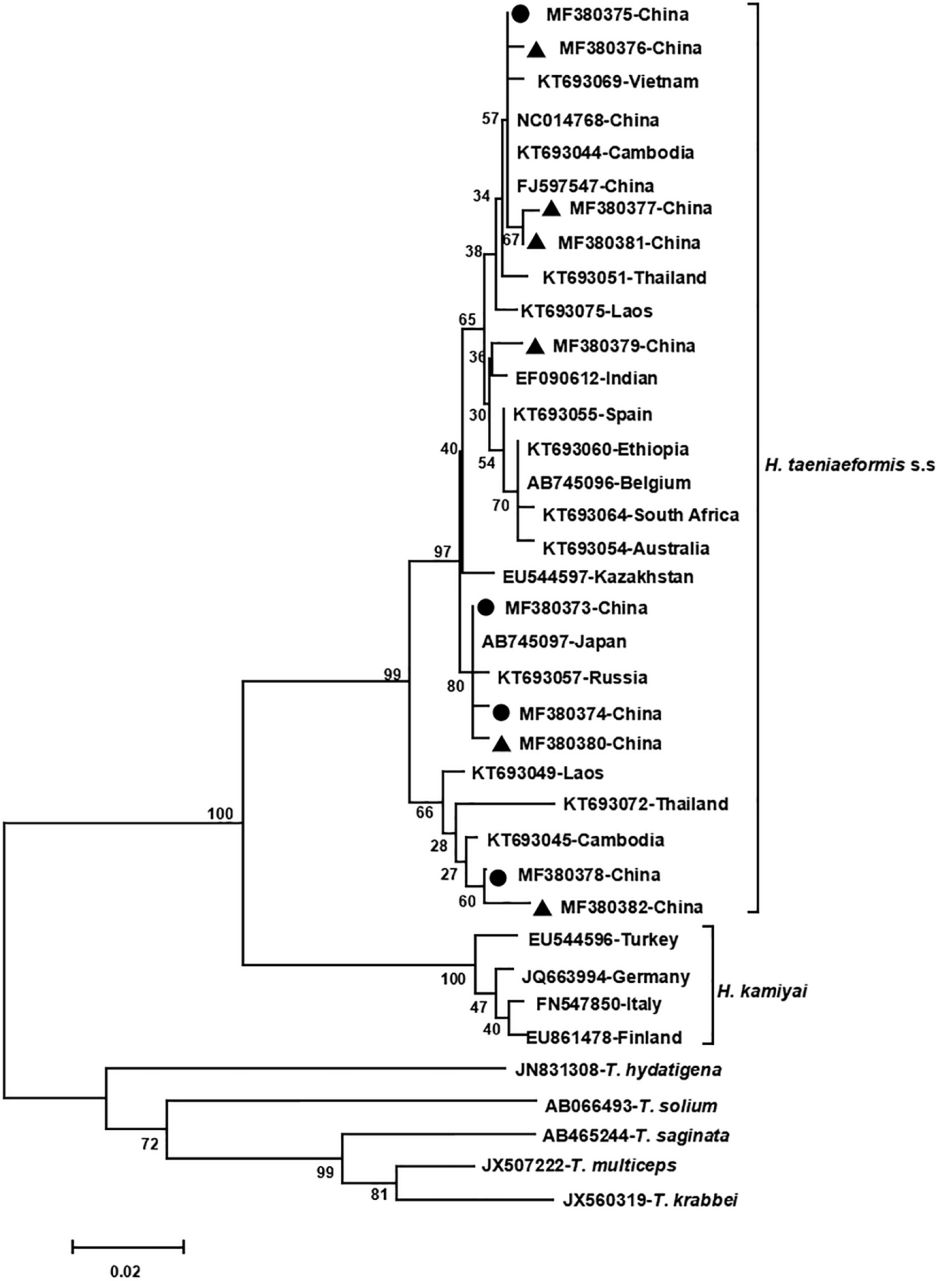
Genetic and geographical relationships of *H. taeniaeformis* s.l. isolates based on a neighbor-joining analysis of the *cox 1* locus. The relationships were inferred by a neighbor-joining analysis of *cox1* gene sequences of *H. taeniaeformis* s.l. isolates from different countries based on genetic distance calculated using the Kimura 2-parameter model. The numbers on the branches are percent bootstrapping values from 1,000 replicates. Each *H. taeniaeformis* s.s. or *H. kamiyai* sequence is identified by its accession number and geographical location (country). Novel and known nucleotide sequences of strobilocercus fasciolaris isolates obtained in the present study are represented by black triangles and black circles, respectively.

**Figure 2:**
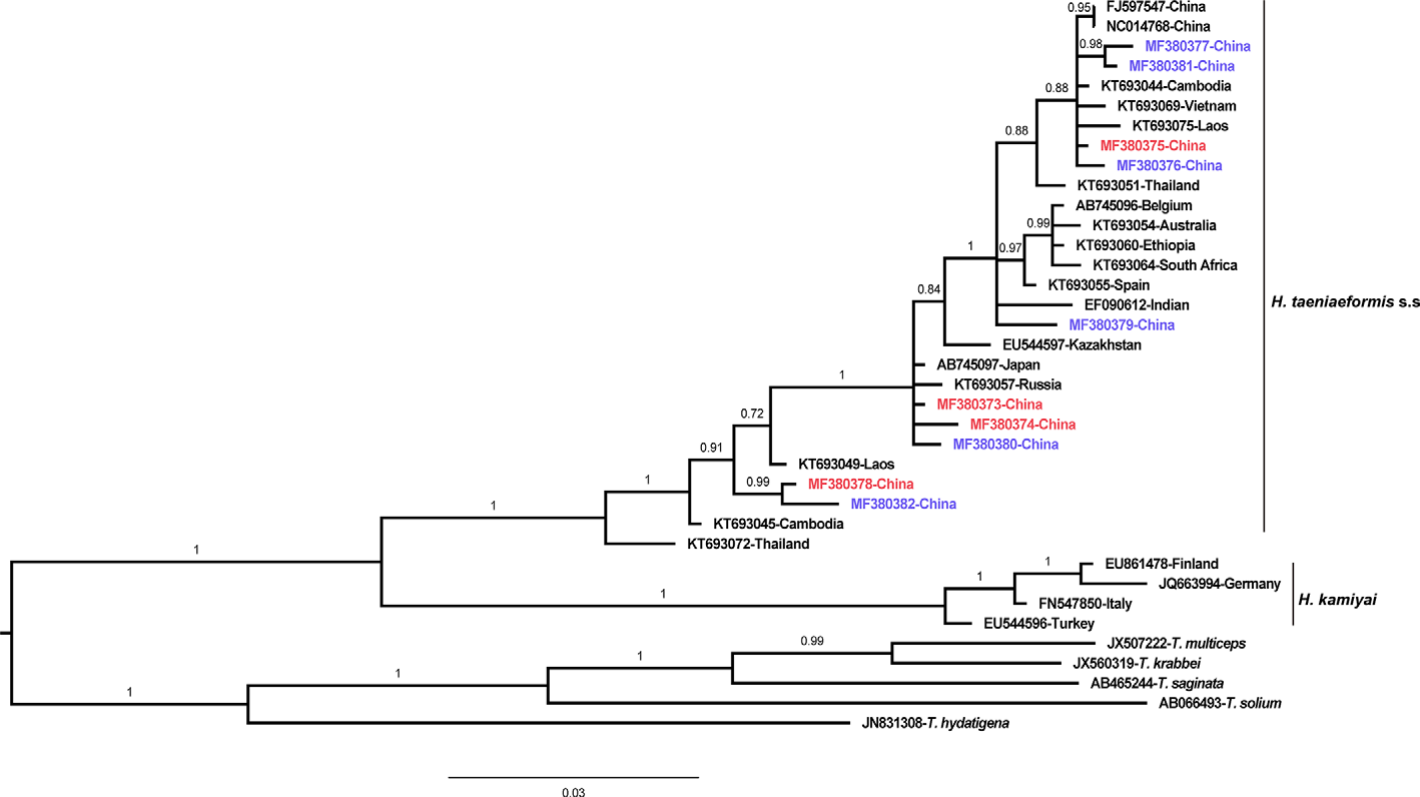
Genetic and geographical relationships of *H. taeniaeformis* s.l. isolates based on a Bayesian inference analysis of the *cox 1* locus. The relationships were inferred by Bayesian inference analysis of *cox1* gene sequences of *H. taeniaeformis* s.l. isolates from different countries based on the Jukes–Canto model. Posterior probability values were produced using MrBbayes. The scale bar displays branch length in units of evolutionary distance. Each *H. taeniaeformis* s.s. or *H. kamiyai* sequence is identified by its accession number and geographical location (country). Novel and known nucleotide sequences obtained in the present study are shown in blue and red, respectively.

**Figure 3:**
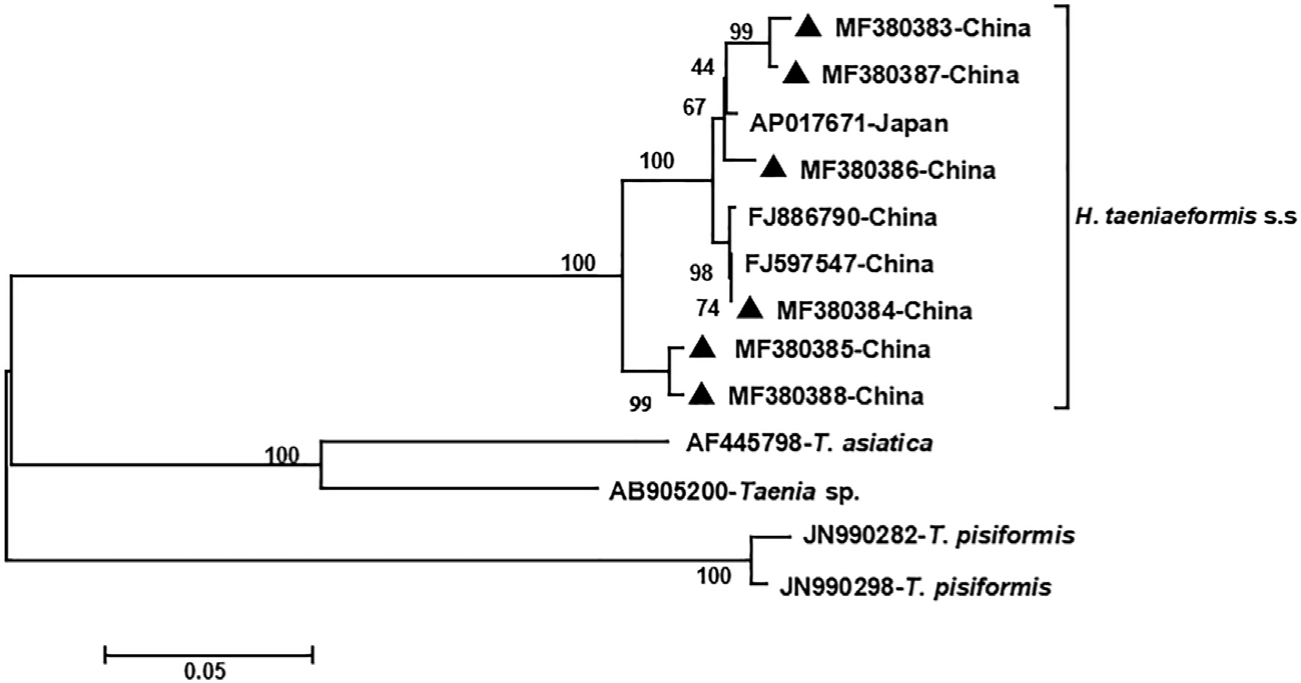
Genetic and geographical relationships of *H. taeniaeformis* s.s. isolates based on a neighbor-joining analysis of the *nad4* locus. The relationships were inferred by a neighbor-joining analysis of the *nad4* gene sequences of *H. taeniaeformis* s.s. isolates from different countries based on genetic distance calculated using the Kimura 2-parameter model. The numbers on the branches are percent bootstrapping values from 1,000 replicates. Each *H. taeniaeformis* s.s. sequence is identified by its accession number and geographical location (country). Novel nucleotide sequences obtained in the present study are represented by black triangles.

**Figure 4:**
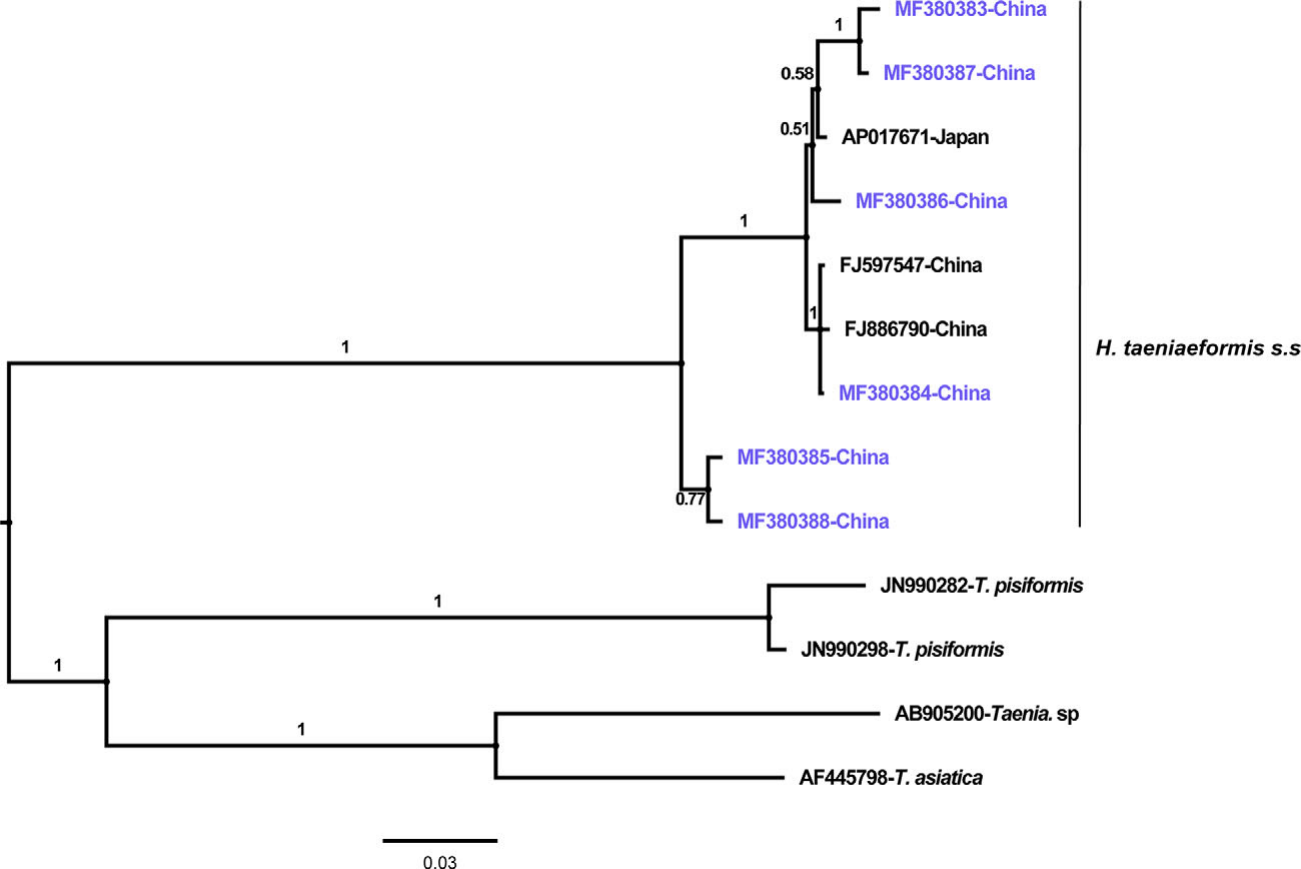
Genetic and geographical relationships of *H. taeniaeformis* s.s. isolates based on a Bayesian inference analysis of the *nad4* locus. The relationships were inferred by Bayesian inference analysis of *nad4* gene sequences of *H. taeniaeformis* s.s. isolates from different countries based on the Jukes–Canto model. Posterior probability values were produced using MrBayes. The scale bar displays branch length in units of evolutionary distance. Each *H. taeniaeformis* s.s. sequence is identified by its accession number and geographical location (country). Novel nucleotide sequences obtained in the present study are shown in blue.

The phylogenetic analyses based on the sequence data of the *cox1* and *nad4* genes revealed that all the haplotypes were grouped with *H. taeniaeformis* s.s., confirming the results of PCR amplification and sequencing. The phylogenetic trees based on neighbor-joining and Bayesian inference analyses were broadly congruent. Regarding the *cox1* locus analysis, all 10 haplotypes belonged to *H. taeniaeformis* s.s.: eight were grouped with those from Europe (Spain, Belgium and Russia), Africa (Ethiopia and South Africa), Oceania (Australia), and Asia (Vietnam, Cambodia, Thailand, Laos, India, Japan, Kazakhstan and China), and two with those from Asia (Cambodia, Thailand, Laos and China). However, several isolates from Europe (Turkey, Germany, Finland and Italy) belonged to *H. kamiyai*, which was recently separated from *H. taeniaeformis* s.l. (Lavikainen et al., 2016) (**Figures 1**, **2**). The *H. kamiyai* isolates were in a single clade, and the results confirmed that *H. kamiyai* is genetically distant from *H. taeniaeformis* s.s. Based on nucleotide sequence comparisons, Catalano et al. found that a *H. taeniaeformis* isolate from a Nile rat (*Arvicanthis niloticus*) in Senegal showed identity to what was described as *H. taeniaeformis* s.s., a lineage that might have originated in Southeast Asia and rapidly invaded Australia, the Americas, Europe and Africa, where it has been identified in Ethiopia and South Africa in *Rattus* spp. (Catalano et al., 2019). Lavikainen et al. (2016) has also speculated that *H. taeniaeformis* probably originated in Asia and has spread worldwide, given its linkage to the Rattini tribe. Regarding the *nad4* locus analysis, all six haplotypes obtained in the present study were grouped with *H. taeniaeformis* s.s. isolates from China (FJ886790 and FJ597547) and Japan (AP017671) (**Figures 3**, **4**). All of these genetic relationships were supported by high Bayesian inference posterior probability values and most of them by moderate-to-high neighbor-joining bootstrap values.

Based on homology analysis of the 10 *cox1* gene sequences identified in the present study, four sequences (MF380373, MF380374, MF380375, and MF380378) have been previously described, while the remaining six (MF380376, MF380377, and MF380379–MF380382) are novel. All of the six *nad4* gene sequences identified in the present study (MF380383–MF380388) are novel. However, only three of the six novel *cox1* gene sequences and five of the six novel *nad4* gene sequence caused amino acid changes relative to their respective reference sequences (the reference sequences were selected on the basis that they had the highest similarity to the representative sequences obtained in the present study) **(** **Table 3** **)**. These findings of novel nucleotide and amino acid sequences might reflect the region-specific genetic characterization of *H. taeniaeformis* s.s. Currently, the significance of these changes at the nucleotide and amino acid levels is unclear. However, *Echinococcus granulosus* research has shown that genetic variation could affect host infectivity, epidemiology and control strategies (Carmena and Cardona, 2014). Similarly, studies on the *Taenia saginata* and *Taenia solium* mt genomes have also demonstrated intraspecific variation that may influence the pathological presentations exhibited in different hosts (Vega et al., 2003; Rostami et al., 2015).

**Table 3:** Homology analysis of the *cox1* and *nad4* loci in strobilocercus fasciolaris isolates from brown rats.

Amplified gene	Accession no. (n) ^a^(no of isolates)		Accession no. ^b^/host/country	Homology (%)	Codon^c/^amino acid (nucleotide Position)^d^
*cox1*	MF380373 (3)	KT693056/leopard cat/Russia; AB221484/brown rat/Japan		100	
	MF380374 (1)	KT693059/striped field mouse/Russia		100	
	MF380375 (1)	FJ597547/cat/China; KT693044/brown rat/Cambodia		100	
	MF380378 (2)	KT693062/leopard cat/Russia		100	
	MF380376 (1)	FJ597547/cat/China		99.8	(T to C)TT/F to L (103)
	MF380377 (1)	FJ597547/cat/China		99.5	(A to G)TT/I to V (310)
	MF380379 (1)	KT693053/small white-toothed rat/Thailand		99.2	
	MF380380 (1)	AB745097/brown rat/Japan		99.8	
	MF380381 (1)	FJ597547/cat/China		99.8	(A to G)TT/I to V (310)
	MF380382 (1)	KT693062/leopard cat/Russia		99.2	
*nad4*	MF380383 (4)	AP017671/unspecific/Japan		98.2	(G to A)CT/(A to T) (355); (T to C)TT/(F to L) (415); AT(A to G)/(I to M) (621)
	MF380384 (5)	FJ597547/cat/China		99.9	
	MF380385 (1)	FJ597547/cat/China		95.9	(A to G)TA/I to V(88); (G to A)AT/D to N (94); AT(A to G)/I to M (330)
	MF380386 (1)	AP017671/unspecific/Japan		98.8	(C to T)CC/(P to S) (124)
	MF380387 (1)	AP017671/unspecific/Japan		98.5	(T to C)TT/F to L (415); AT(A to G)/I to M (621)
	MF380388 (1)	AP017671/unspecific/Japan		95.9	(A to G)TA/I to V (88); (C to T)C(C to T)/P to S (124; 126); AT(A to G)/I to M (330); A(C to T)A/T to I (386)

^a^Accession no. of the representative sequences obtained in the present study.

^b^Accession no. of the reference sequences, which had the highest similarity with the representative sequences obtained in the present study.

^c^The nucleotide change (in brackets for each codon) represents the change from the reference sequence to the representative sequence obtained in the present study.

^d^Nucleotide position numbers according to the representative sequence, with the beginning of the coding region being position no. 1.

## Conclusion

This is the first report on the genetic characterization of strobilocercus fasciolaris in brown rats in China. High genetic heterogeneity was found across the 13 identified isolates: 10 haplotypes (intraspecific variation: 0.3%–4.4%) at the *cox1* locus and six haplotypes (intraspecific variation: 0.6%–4.7%) at the *nad4* locus. Based on phylogenetic and homology analyses, all 13 isolates belonged to *H. taeniaeformis* s.s. The findings of novel nucleotide and amino acid sequences might reflect the endemic genetic characterization of strobilocercus fasciolaris. The molecular data will be useful to further explore the biological and epidemiological significance of intraspecific variation within *H. taeniaeformis* s.s.

## Data Availability Statement

The datasets presented in this study can be found in online repositories. The names of the repository/repositories and accession number(s) can be found below: https://www.ncbi.nlm.nih.gov/genbank/, MF380373–MF380382 (*cox1* gene) and MF380383–MF380388 (*nad4* gene).

## Ethics Statement

The animal study was reviewed and approved by Research Ethics Committee and the Animal Ethical Committee of Harbin Medical University. Procedures involving animals were strictly conducted according to the Chinese Laboratory Animal Administration Act of 1998.

## Author Contributions

AL and WZ conceived and designed the study. FY, FZ, and YZ performed the study and analyzed the data. FY wrote the first draft of the manuscript. WZ, YW, and KZ provided strategic advice and assisted with editing the manuscript. All authors contributed to the article and approved the submitted version.

## Funding

This work was financially supported by the Heilongjiang Province Education Bureau of Foundation (No. 12531266) and by the Natural Science Foundation of Heilongjiang Province of China (No. H2017006). The funding sponsors had no role in study design, data collection and analysis, decision to publish or preparation of the manuscript.

## Conflict of Interest

The authors declare that the research was conducted in the absence of any commercial or financial relationships that could be construed as a potential conflict of interest.
